# The importance of endpoint selection: How effective does a drug need to be for success in a clinical trial of a possible Alzheimer’s disease treatment?

**DOI:** 10.1007/s10654-018-0381-0

**Published:** 2018-03-23

**Authors:** Stephanie Evans, Kevin McRae-McKee, Mei Mei Wong, Christoforos Hadjichrysanthou, Frank De Wolf, Roy Anderson

**Affiliations:** 1 Department of Infectious Disease Epidemiology, School of Public Health, Imperial College London, London, UK; 2 Janssen Prevention Center, Leiden, The Netherlands

**Keywords:** Alzheimer’s disease, Clinical trials, Longitudinal data analysis

## Abstract

To date, Alzheimer’s disease (AD) clinical trials have been largely unsuccessful. Failures have been attributed to a number of factors including ineffective drugs, inadequate targets, and poor trial design, of which the choice of endpoint is crucial. Using data from the Alzheimer’s Disease Neuroimaging Initiative, we have calculated the minimum detectable effect size (MDES) in change from baseline of a range of measures over time, and in different diagnostic groups along the AD development trajectory. The Functional Activities Questionnaire score had the smallest MDES for a single endpoint where an effect of 27% could be detected within 3 years in participants with Late Mild Cognitive Impairment (LMCI) at baseline, closely followed by the Clinical Dementia Rating Sum of Boxes (CDRSB) score at 28% after 2 years in the same group. Composite measures were even more successful than single endpoints with an MDES of 21% in 3 years. Using alternative cognitive, imaging, functional, or composite endpoints, and recruiting patients that have LMCI could improve the success rate of AD clinical trials.

**Electronic supplementary material:**

The online version of this article (10.1007/s10654-018-0381-0) contains supplementary material, which is available to authorized users.

## Introduction

All cause dementias are one of the world’s leading health concerns. In the absence of effective therapies, is it estimated that the number of people with dementia will reach 131.5 million by 2050. Alzheimer’s disease (AD) is the most common form of dementia accounting for 50–75% of all case that typically affect the older age groups [1]. AD is a neurodegenerative condition characterised by a progressive decline in cognitive function, accompanied by changes in the concentrations of certain proteins (e.g. Amyloid_1-42_ (A$\upbeta_{{1{-}42}}$) and tau) in cerebral spinal fluid (CSF), and changes in the brain that can be picked up by scanning technologies such as Magnetic Resonance Imaging (MRI) [2].

There is currently no treatment or cure, and in 2016 the Office for National Statistics reported that AD had overtaken cardiovascular disease to become the leading cause of death in England and Wales [3]. Unlike cardiovascular disease where 41 drugs have been approved by the U.S Food and Drug Administration (FDA) since 2002, only five drugs that provide short-term symptomatic relief and have no preventative or curative activity, have been marketed in the AD therapy area since 1984. No new drugs have been approved by the FDA since 2002 [4].

The high attrition rate in clinical trials (CTs) of possible AD therapies has been attributed to a number of factors including inadequate target selection due to the uncertainty surrounding the biological mechanisms behind disease development [5], and the true efficacy of a treatment being masked by the variance in the endpoint employed [6]. The nature of AD as a slowly developing disease over many decades means that the timespan of a CT, typically less than 2 years [7] could be too short for an effect to be detected.

In their 2016 draft guidelines for clinical investigation of medicines for the treatment of AD, the European Medicines Agency (EMA) states that efficacy in an AD CT should be measured by a cognitive, functional and clinical endpoint when considering patients with established AD [5]. However, in patients with less severe disease the guidelines are more ambiguous. In patients with prodromal AD or mild cognitive impairment (MCI), they recommend the use of two co-primary endpoints assessing cognition and function, and in preclinical AD patients they state that there is no gold standard for assessment. The FDA guidelines also state that CTs in on AD should use a co-primary outcome measure approach in which a drug demonstrates efficacy on both a cognitive and a functional or global assessment scale [8], suggesting the use of a composite cognitive and functional score as a suitable tool for assessment in early disease and giving CDR-SB as an example of such an endpoint. However, they also state that they would consider approving isolated cognitive measures as endpoints in trials where patients are in a preclinical AD stage. Biomarkers are not currently accepted as endpoints but the FDA will consider them for approval as either primary or secondary outcome measures if sufficient evidence can be provided [8]. Despite these guidelines, the Alzheimer’s Disease Assessment Scale-cognition subscale (ADAS-Cog) [9] is still the most widely used general cognitive measure in AD CTs [10]. This is despite concerns that ADAS-Cog may underestimate changes in and differences between patients given the drug and those in the control group. These concerns are particularly pertinent when dealing with patients with MCI or early AD [11, 12], or when the length of the trial is less than 18 months [6, 13].

The Alzheimer’s Disease Neuroimaging Initiative (ADNI) is a consortium of universities and medical centres in the United States and Canada that have formed a longitudinal observational cohort study to identify new imaging biomarkers measuring AD progression [14]. A range of cognitive, biomarker, and functional data has been recorded.

We aim to investigate whether there are measures in any of these three groups that could be used as endpoints to increase the probability of success in an AD preventative CT. As it is not feasible for us to assess the potential of every measure recorded in ADNI as an endpoint, we have selected a small subset of measures that we believe are appropriate for demonstrating our case, that ADAS-Cog may not be the most suitable endpoint for AD trials. Using a formula described by [15], we have calculated the minimum detectable effect size (MDES), defined as the absolute change from baseline that lies outside of the sum of the type I and type II error levels for a standard Z-test, for a selection of measures from each of the three groups in ADNI. We report the required treatment efficacy as the percentage by which the actual change from baseline in an untreated would have to be reduced by to bring the value of each measure back within a non-detectable region from the baseline value, for different time points in the study.

## Methods

### Dataset

For our analysis we used the ADNI study (adni.loni.usc.edu) that was launched in 2003 as a public–private partnership, led by Principal Investigator Michael W. Weiner, MD. The primary goal of ADNI has been to test whether serial MRI, PET, other biological markers, and clinical and neuropsychological assessment can be combined to measure the progression to MCI and to early AD. For up-to-date information, see www.adni-info.org. The dataset used was downloaded on 31st October 2016 from the ADNI server. 106 individuals with a “subjective memory concern” (SMC) diagnosis at baseline were excluded from the analyses. Due to the decreasing sample size in each of the four cognitive groups over time, data from visits more than 6 years after baseline was discarded for all individuals on the grounds that there was insufficient data in any of baseline diagnostic groups after this time point (Table S1). The MDES was calculated for a selection of cognitive, biological, and functional measures. These variables, along with their availability in the ADNI dataset are shown in Table 1.

**Table 1:** Availability of measures of interest in ADNI

ADAS11	ADAS13	MMSE	MOCA	Hip Vol	WB Vol	CDR-SB	FAQ
5.33	5.17	5.50	0.83	3.67	3.83	5.50	5.50

Average number of measurements per individual in ADNI for the Measures of of interest in this study. *Hip Vol* hippocampal volume, *WB Vol* whole brain volume

### Cognitive markers in ADNI

Potential cognitive endpoints that have been recorded in the ADNI study include the mini-mental state evaluation score (MMSE), Montreal cognitive assessment (MoCA), and a more comprehensive version of the ADAS-Cog measure [16].

The ADAS-Cog was designed specifically to identify AD in a CT [9]. The ADNI study contains two variants of ADAS-Cog that score patients using either 11 or 13 subscales [17] allowing participants to score a maximum of 70 points (ADAS-Cog-11) or 85 points (ADAS-Cog-13) respectively, with lower scores indicating better cognitive function.

The MMSE was developed to evaluate the cognitive performance of psychiatric patients as an alternative to other cognitive scoring tests that were lengthy to administer [18]. Scores range from 0 to 30 with a higher score indicative of better cognitive function and cut-off points are typically defined as follows; ≥ 24 Cognitively Normal (CN), 18-23 MCI, ≤ 18 AD [19]. MMSE is often recorded as a secondary endpoint in AD-CTs but is not commonly used as a primary endpoint.

The MoCA scoring system was developed to screen MCI individuals who have MMSE scores of 24 or higher thus are considered to be CN based on MMSE alone [20]. Like MMSE, MoCA is often listed as a secondary endpoint in CTs.

### Magnetic resonance imaging markers in ADNI

One of the major goals of the ADNI study is to develop standardised imaging techniques to help create uniform standards for acquiring longitudinal magnetic resonance imaging (MRI) data [21]. As such, ADNI database contains several MRI measurements including hippocampal and whole brain volume that are thought to be useful for the classification of cognitively impaired individuals into an AD or MCI subset. For details of how the MRI volumes are calculated, see [22, 23].

Hippocampal volume atrophy has long been associated with disease progression in AD [24, 25] and it has been suggested that hippocampal atrophy could be used as a surrogate marker for efficacy in an AD CT [22, 26].Whole brain atrophy has also been strongly associated with cognitive decline [27], with rates of atrophy typically being higher the further down the AD disease trajectory a patient lies.

### Functional markers in ADNI

A decline in the ability to perform daily activities such as handling finances, shopping, using the telephone, and managing medication is an important factor in diagnosing AD using the Diagnostic and Statistical Manual (DMS-VI). There are several methods to assess functional capabilities recorded in ADNI, including the Clinical Dementia Rating Sum of Boxes (CDRSB), and Functional Activities Questionnaire (FAQ).

The CDRSB is a composite score assessing both cognitive function and daily living activities. The score ranges from 0 to 18, and is calculated by summing over scores in six domains including memory, orientation, judgment/problem solving, community affairs, home and hobbies, and personal care, with higher scores indicative of more severe disease [28].

The FAQ measures activities such as preparing meals and managing personal finances [29]. The FAQ score ranges from 0 to 30 and can be used to differentiate those with mild cognitive impairment and mild Alzheimer’s disease [30].

### Composite measures calculated from ADNI

Although not yet common in CTs, several composite measures of AD-related decline have been proposed in the literature. We have calculated three of these measures using the ADNI dataset, namely the AD Composite Score (ADCOMS) [31]. Preclinical Alzheimer’s Cognitive Composite (PACC) [32], and a five item composite proposed by Huang et al. [33]. ADCOMS consists of four ADAS-Cog items, two MMSE items, and six CDR-SB items, and is designed to provide improved sensitivity for measuring cognitive decline in amnestic MCI, prodromal AD, and in mild AD dementia. The PACC was designed to estimate decline in preclinical AD groups that A$\upbeta_{{1{-}42}}$ positive. This score consists of the Total Recall score from the Free and Cued Selective Reminding Test (substituted with the Delayed Recall from the ADAS-cog test in ADNI, as advocated by [32]), the Delayed Recall score on the Logical Memory IIa subtest, the Digit Symbol Substitution Test score, and the total MMSE score. In the construction of the PACC, all of these measures are standardised by dividing by the baseline standard deviation, before summing to generate an overall score. The third composite developed by Huang et al., is the sum of Word Recall, Delayed Recall and Orientation scores from the ADAS-cog, along with CDR-SB and FAQ scores. It was designed to improve detection of decline in A$\upbeta_{{1{-}42}}$ positive MCI individuals.

### Minimum detectable effect size calculations

For a treatment effect to be statistically significant at the α level with a one-tailed hypothesis test (or at the α/2 level with a two-tailed test), the estimate of the mean must fall to the right of the α-level critical value. Further, to have a probability 1 − β of detecting a treatment effect, the mean treatment effect must lie a distance greater than or equal to 1 − β-level critical value to the right of the critical value under the null hypothesis where β represents the level of statistical. The MDES that can be statistically identified between two populations in a randomised trial is therefore.

1 $$MDES = \left( {v_{\alpha } + v_{1 - \beta } } \right)\sqrt[]{{\frac{{\sigma^{2} }}{np(1 - p)}}},$$

where $v_{\upalpha}$ is the α-level critical value of the distribution used in the hypothesis test, $v_{{1 -\upbeta}}$ is the 1 − β-level critical (typically 80%), *σ* is the pooled standard deviation of the trial endpoint, *n* is the total number of individuals in the trial at the time point under consideration, and *p* is the proportion of individuals in the treatment group [15].

## Results

### Detecting an effect in cognitive markers

**Fig. 1:**
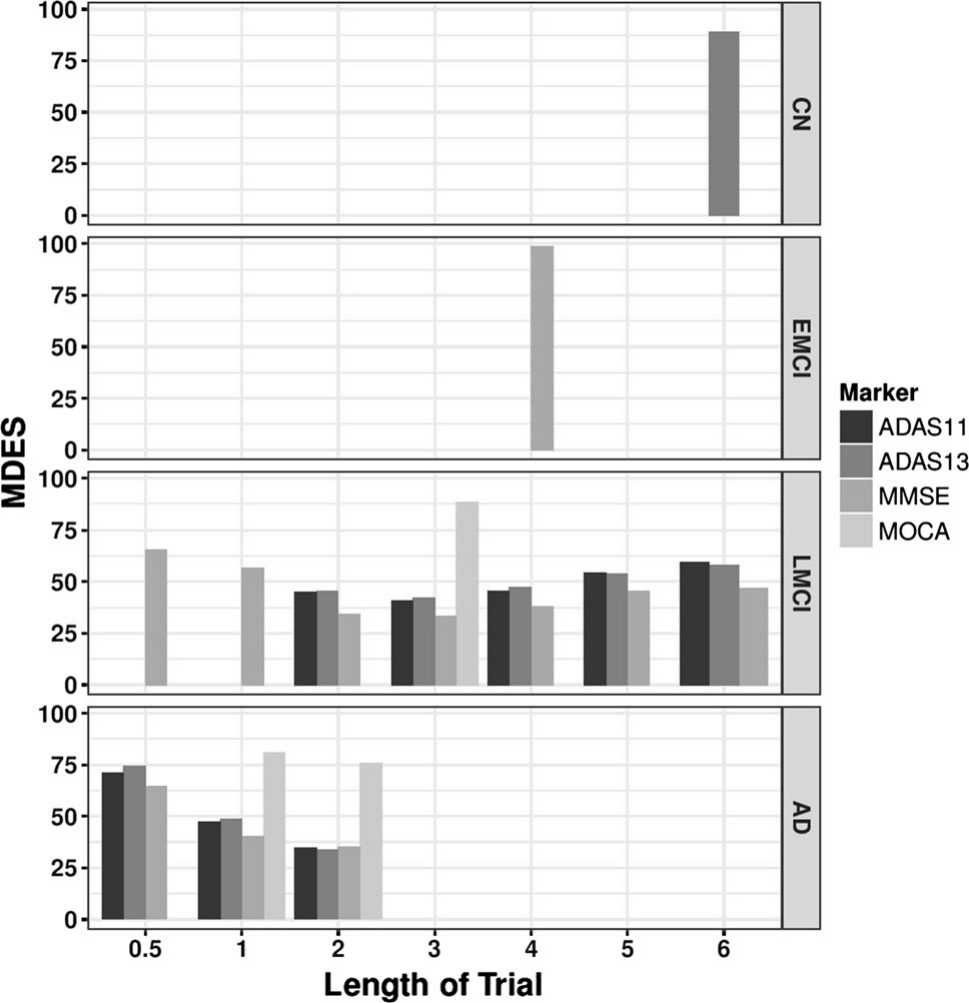
Minimum detectable effect size in four measures of cognition. MDES was calculated for ADAS-Cog 11, ADAS-Cog13, MMSE, and MOCA over 6 years from baseline in the ADNI study. Missing bars indicate a non-detectable effect size, or time points where there were less than 100 people thus have been excluded from the analysis

The MDES was calculated for four cognitive markers, ADAS-Cog11, ADAS-Cog13, MMSE and MoCA (Fig. 1). The data from ADNI suggest that a CT that uses ADAS-Cog-11 as an end point would be unable to detect a treatment effect within 6 years if the patients in the trial were either CN or had early MCI (EMCI) at baseline, even if the treatment acted instantly and with 100% efficacy. If the baseline population was composed of individuals diagnosed with late MCI (LMCI), an effect could be detected within 2 years if a treatment slowed the increase in ADAS-Cog-11 by at least 45%. In this LMCI population, the MDES increases at later time points. The group that was AD at baseline had the smallest MDES, and an effect of 35% could be detected in 2 years (Fig. 1).

Similarly to ADAS-Cog11, if the endpoint of a CT with baseline demographics the same as in the ADNI database was taken to be ADAS-Cog13, it would be difficult to detect an effect in a CN population with treatment efficacy of 100% detectable in a 6 year trial, and impossible to detect an effect in a population of patients with EMCI. The LMCI population gave the highest chance of success with a 45% efficacy detectable within 2 years, although as with ADAS-Cog11, increasing the length of the trial past 4 years had a negative effect on the MDES. A 35% effect size could be identified in a 3 year trial. In AD group, the MDES was 38%.

Using MMSE as an endpoint in a CT, no effect will be detected in a 6 year trial if the population is CN at baseline which is unsurprising given that MMSE was not designed to be used in CN individuals. In a population with EMCI at baseline, a drug would have to have 100% efficacy for an effect to be detected. However, using MMSE as an endpoint allows an effect to be detected in the LMCI group at an earlier time point than either of the ADAS-Cog scores, with a treatment effect that slowed the decline in MMSE score by 60% detectable within 1 year, and 35% by 2 years. Again, a lower effect can be detected in the AD group at 1 year (35%) but there is no advantage to using an AD group, over a set of patients with LMCI in a 2 year trial (Fig. 1).

The MoCA scores did not reveal a detectable effect in any diagnostic group within 6 years (Fig. 1).

### Detecting an effect in MRI markers

**Fig. 2:**
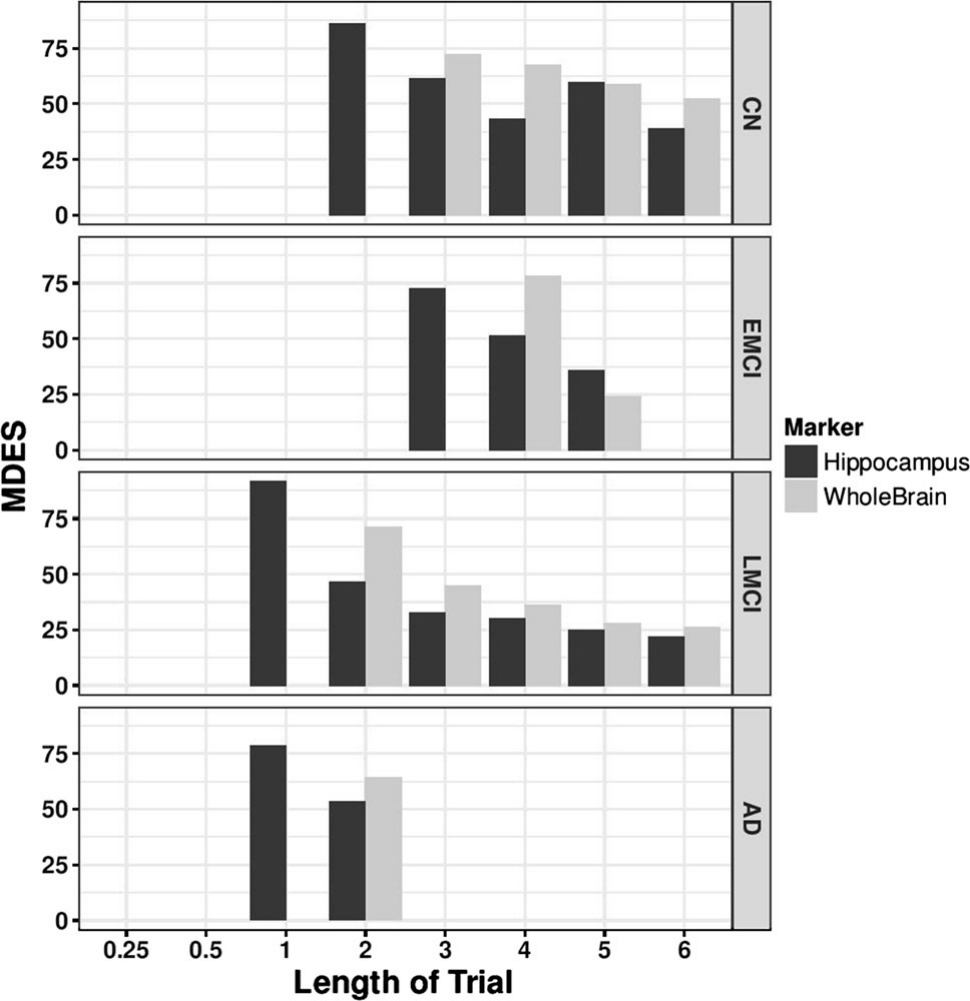
Minimum detectable effect size in MRI measures. MDES was calculated for hippocampal atrophy (black) and whole brain atrophy (grey) over 6 years from baseline in the ADNI study. Missing bars indicate a non-detectable effect size, or time points where there were less than 100 people thus have been excluded from the analysis

Hippocampal and whole brain atrophy could be considered to be targets in a CT, however here we consider their utility as endpoints in CTs where they are not directly targeted, thus we estimated the MDES using hippocampal atrophy, and whole brain volume (Fig. 2).

An effect of altering the rate of hippocampal atrophy can be detected in all diagnostic groups within 3 years from baseline. The CN group demonstrated a detectable therapy effect size of 86% in 3 years but this improves to being able to detect a 39% effect in a 6 year trial. In the EMCI group, a therapy effect of 72% can be detected in 3 years, and this improves to a detectable therapy effect of 36% in a trial lasting 5 years. The LMCI group has the smallest MDES, with an efficacy of 92% can be detected after 1 year, 46% by 2 years and less than 30% from trials of 3 or more years. In the AD group, slowing the decline in hippocampal atrophy could only be detected at one and 2 years (79 and 53% respectively).

When taking whole brain atrophy as an endpoint, no effect can be detected in the CN population until 3 years (72%) and the minimum effect size that can be detected is 59% at 6 years. The EMCI group has an MDES of 78% at four years but this improves to 28% by 5 years. In the LMCI group, an effect size of 71% can be detected within 2 years, and this is improves to 28% in a trial of more than 3 years.

### Detecting an effect in dementia rating or functional activities

**Fig. 3:**
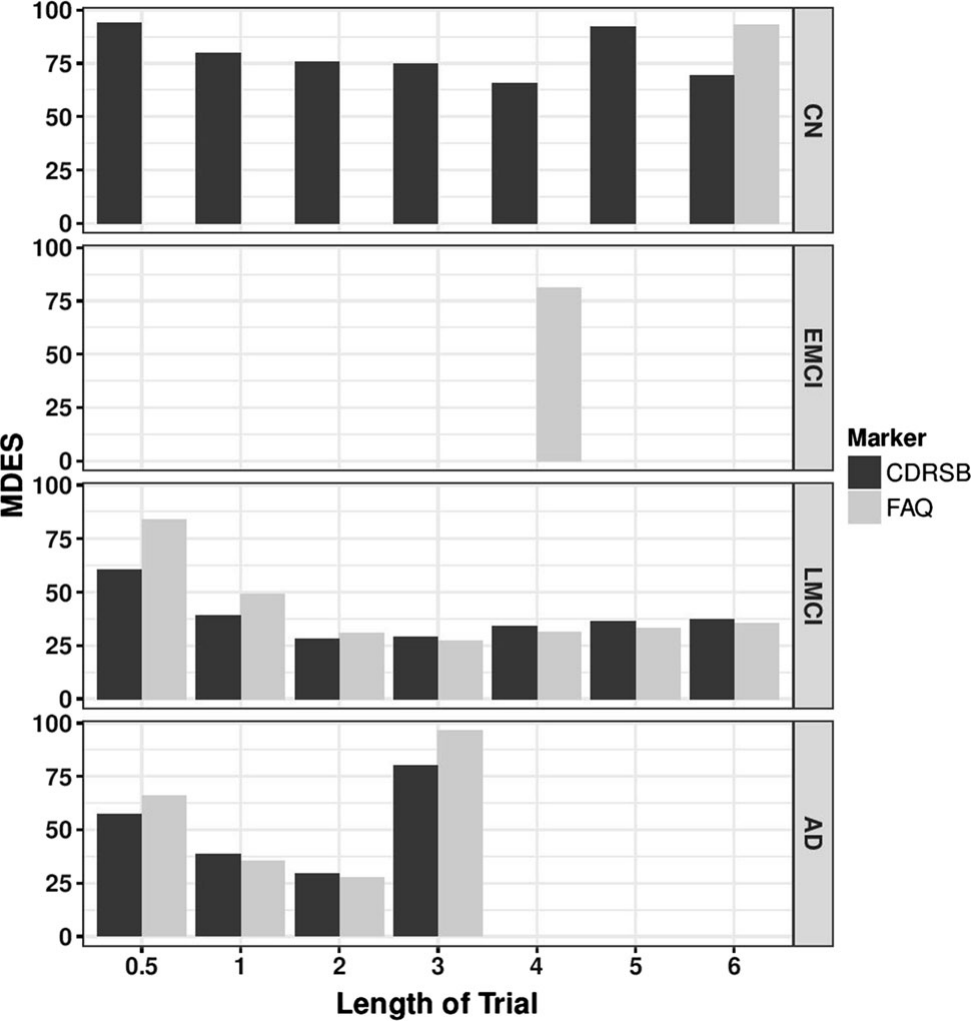
Minimum detectable effect size in CDRSB and FAQ. MDES was calculated for CDRSB (black) and FAQ (grey) over 6 years from baseline in the ADNI study. Missing bars indicate a non-detectable effect size, or time points where there were less than 100 people thus have been excluded from the analysis

We calculated the MDES for the clinical dementia rating sum of boxes (CDRSB), and the functional activity questionnaire (FAQ) scores (Fig. 3). The CDRSB had a detectable effect in all groups except for EMCI. In the CN group, the minimum MDES in the first 6 years (66%) occurred at 4 years, but an effect was detectable at all time points in this group. In the LMCI group, an effect of 29% could be detected in a 2 year trial. This effect size did not change significantly as the length of the trial increased. In the AD group, the MDES was also 29%, again occurring after 2 years.

The FAQ endpoint gave similar results to CDRSB in the more severe populations but had a higher MDES in the CN population, with an effect of 90% only detectable after 6 years. In the EMCI population, an effect size of 81% would be detectable in a trial lasting 4 years. The LMCI and AD populations had a MDES of around 30%.

### Detecting an Effect in Composite Endpoints

**Fig. 4:**
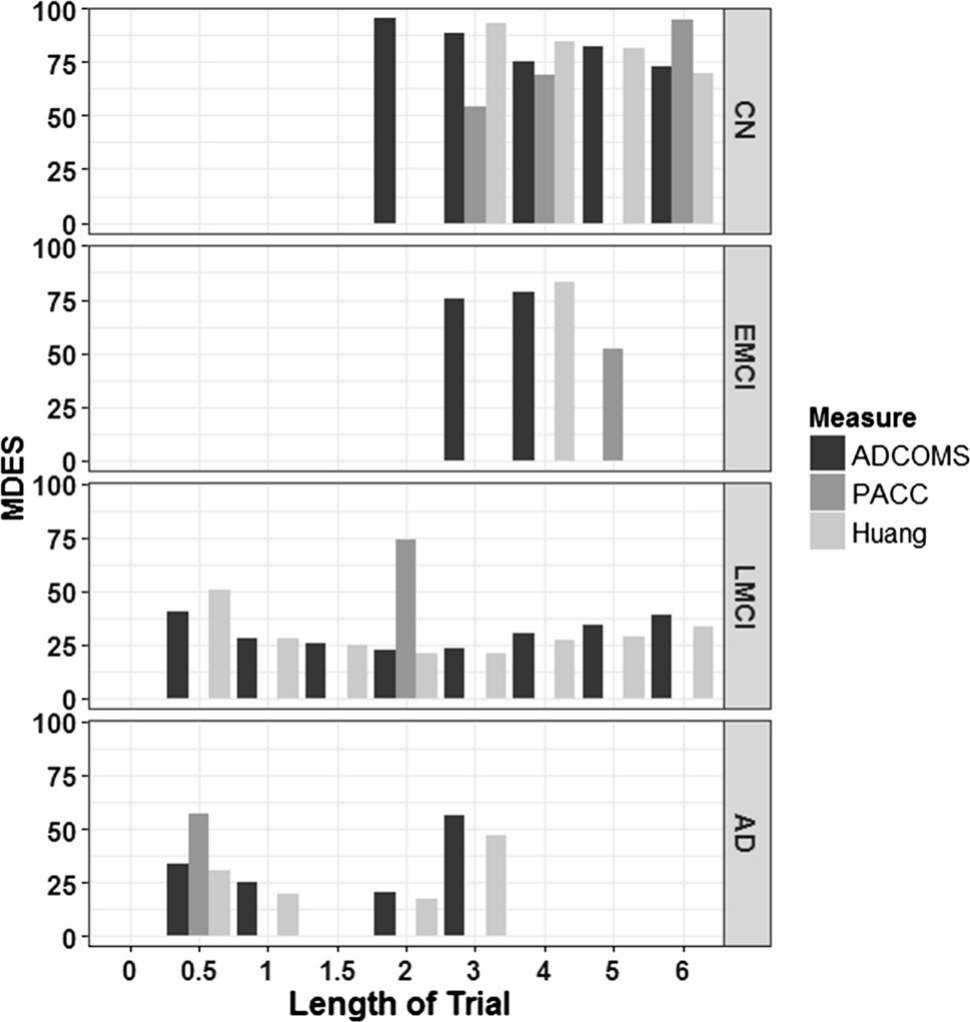
Minimum detectable effect size in composite endpoints. MDES was calculated for ADCOMS, PACC, and the measure generated by Huang et al. [33] over 6 years from baseline in the ADNI study. Missing bars indicate a non-detectable effect size, or time points where there were less than 100 people thus have been excluded from the analysis

We calculated the MDES for three previously published composite endpoints, ADCOMS, PACC, and another by Huang et al. [33] (Fig. 4). Using the ADCOMS measure allows an effect of 40% to be detected by 6 months in the LMCI population, and an effect of 33% at the same time point in the group that had AD at baseline. The minimum effect that can be detected with the ADCOMS measure is 22% by 2 years in the LMCI population, or 20% by 2 years in the AD group. To detect an effect in either the CN or EMCI populations using ADCOMS as an outcome measure the effect of the treatment would have to be at least 75% and the trial would need to run for 4 years (CN) or 3 years (EMCI).

The PACC is most successful in detecting a change in the CN population with an effect size of 51% being identifiable by 3 years. It is the least successful endpoint for detecting change in the LMCI and AD groups.

The composite proposed by Huang et al. [33] allows an effect of 21% to be identified in the LMCI group by 2 years. It is slightly more successful than ADCOMS in determining a change in the AD group, and at later time points in the LMCI group.

### Length of Trial on MDES

**Fig. 5:**
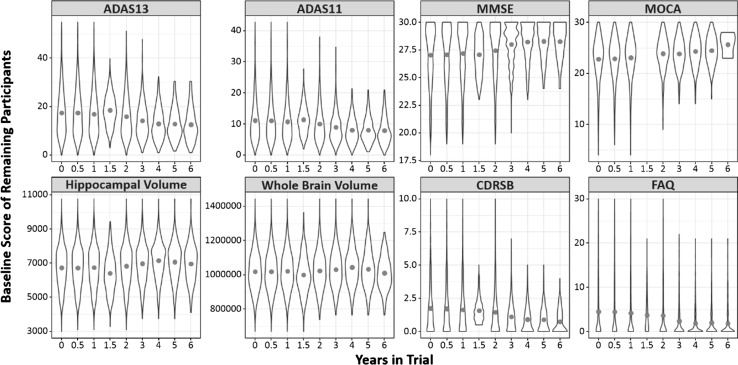
Individuals retained in the study past 3 years are less abnormal at baseline. Violin plots show the distribution of the baseline values of the measures used in this study for the individuals retained at each time point. Red points indicate the mean baseline value of each measures over time

For almost all of the endpoints that we considered, increasing the length of the trial from 0.5 to 3 years decreases the MDES, thus improving the likelihood of a treatment being successful. However, after 3 years, the MDES of a change in score/marker level from baseline either stays approximately the same level, or increases. Figure 5 shows the distribution of the baseline markers for those individuals still involved with the study at each time point in the ADNI study. For all cognitive and functional endpoints, individuals who remain in the study after 3 years have less abnormal baseline values of these measurements, and are therefore expected to decline at a slower rate. However, there is not a significant change in the variability of the baseline values for these individuals. It would therefore be less likely that a treatment effect could be detected in this population in a CT where change from baseline in a treatment versus control group using one of these endpoints was the outcome of interest.

## Discussion

In this study we assessed the MDES of potential cognitive, imaging, functional and composite clinical trial endpoints when compared to baseline measures using the ADNI study (Table 2). We have demonstrated that several single endpoints may be better than the ADAS-Cog, that is widely used and can be considered as standard, for detecting a treatment effect in patients that have either LMCI or AD at baseline, namely a decline in MMSE, hippocampal atrophy, whole brain atrophy, an increase in CDRSB, and an increase in FAQ. The composite endpoints ADCOMS and that proposed by Huang et al. [33] are also more sensitive than ADAS-Cog in an LMCI group. In addition to the work presented here, we explored the MDES using CSF markers but found no detectable effect within 6 years.

**Table 2:** Summary of results of MDES calculations

Measure	Population	MDES	Time
ADAS-Cog11	CN		
	EMCI		
	LMCI	41%	3 years
	AD	35%	2 years
ADAS-Cog13	CN	89%	6 years
	EMCI		
	LMCI	42%	3 years
	AD	34%	2 years
MMSE	CN		
	EMCI	99%	4 years
	LMCI	34%	3 years
	AD	35%	2 years
MOCA	CN		
	EMCI		
	LMCI	89%	3 years
	AD	76%	2 years
Hippocampus	CN	35%	6 years
	EMCI	34%	5 years
	LMCI	21%	6 years
	AD	54%	2 years
Whole brain	CN		
	EMCI		
	LMCI		
	AD		
CDRSB	CN	66%	4 years
	EMCI		
	LMCI	28%	2 years
	AD	30%	2 years
FAQ	CN	96%	6 years
	EMCI	81%	4 years
	LMCI	27%	3 years
	AD	28%	2 years

Empty rows indicate no effect can be detected over a 6 year trial

The FDA has provided new draft guidelines for clinical trial endpoints in patients at different stages of disease ranging from stage 1, where patients have pathological abnormalities to stage 4 with severe dementia, stating that cognitive endpoints are appropriate for patients in stage 1 or 2 of the disease (pathological symptoms but no or little cognitive complaints), but that an integrated scale assessing both function and cognition such as the composites examined in this work would be an appropriate, and acceptable endpoint in patients with stage 3 and 4 of the disease [34].

After conducting this study, we would suggest that a potentially effective trial design would involve targeting an LMCI or AD population for at least 2 years and using functional scores such as CDRSB as a single endpoint, or ADCOMS as a composite. If a single cognitive endpoint was to be used, we would suggest using MMSE over ADAS-cog since a lower efficacy treatment effect can be identified using this measure. Further, an effect can be detected earlier using MMSE in an LMCI population.

The issue of detectable effect sizes in AD CTs is particularly pertinent following the recent failures of promising drugs including Solanezumab in a trial using change in ADAS-cog at 80 weeks as a primary endpoint, with patients selected on diagnostic group (mild AD), Aβ status and MMSE at baseline. Using the methodology presented here, we estimate that the MDES in this trial would have been 4.07 points in ADAS-cog, far above the change of 0.8 in the trial, but had the endpoint been chosen to be one of the composite scores, it is possible that a significant effect could have been detected.

Previous work has focused on estimating required sample sizes for a trial to be successful using variety of endpoints but with predefined therapy efficacies [35] (reviewed by [36]). While these analyses have provided insight into sample sizes required to detect an effect of a treatment with 25% efficacy, the numbers produced are often infeasible for CT situation, and such work does not provide evidence as to the size of the effect that can be detected when the population size drawn from an acceptable CT design. By using longitudinal patient data from the ADNI study, we have estimated the most efficient single and composite measures for detecting a clinical effect using change-from-baseline over 6 years, for four baseline diagnosis groups. The advantages in studying effect size in this manner are two-fold. As well as being able to make inferences about ideal populations and time-spans for clinical trials, we have been able to account for the effect of withdrawal of participants from the study on the MDES. This effect is seen most strongly when considering the cognitive scores as endpoints (Fig. 1) but also occurs with functional and composite measurements (Figs. 3, 4). In the LMCI group, the MDES increases after 3 years, meaning that in a trial of three or more years where all participants start as LMCI, we are less likely to detect an effect than in a shorter trial. There are two possible reasons for this, firstly, the sample size reduces year upon year (Table 1), but this can be accounted for by taking a larger starting population. However, on average the baseline measurements of the patients that are retained in the trial past 3 years are less abnormal than for those that withdraw. This is an artefact created by using the mean change from baseline methodology that is commonly adopted in AD CTs [35], because those with worse baseline scores, who are expected to progress to AD at a faster rate, are more likely withdraw from the trial so those individuals that are left in the trial at later time points had, on average, higher cognitive or functional scores at baseline, and have a lower rate of decline over time. The effect of removing 10% of the patients with high ADAS-cog 1 or 13 at baseline (defined as those with a score greater than 1 standard deviation away from the mean) increases the MDES at 3 years by 1 and 0.5% respectively in the LMCI. However, given that ADNI is a more homogeneous population than a general LMCI patient group, this effect could be higher in a clinical trial situation and needs studying further using a larger, or more regular population. This effect does not appear when considering the MRI markers, suggesting that the rate of brain atrophy is not dependent on the baseline measurement (Fig. 2).

There are several limitations to this study. Firstly, we only compared mean change from baseline, not the rate of change in measurements over time as has been suggested by some [37, 38]. However, the FDA have not reached a conclusion as to whether the comparison of the rate of change of a marker between treatment and control groups could act as a sole endpoint in a CT [8] thus mean change from baseline is the most clinically relevant comparison at this point in time. Furthermore, we have calculated the MDES assuming that any treatment would act immediately from baseline with the specified effect, and that the effect would be linear over time. However, a simple addition can take account of pharmacokinetics and pharmacodynamics if the drug efficacy required to achieve a detectable effect for a non-linear treatment effect is known. The results presented here are generalizable to trials in which patient populations are classified in the same way as in the ADNI dataset. It is possible that the MDES in the markers described here (most notably hippocampal volume, but also ADAS-cog to some extent), could be underestimated within the four baseline demographic groups in ADNI than in such cognitive subgroups in the general population. It should also be noted that treatments targeting vascular risk factors or conditions such as hypertension or diabetes may provide improvements in different cognitive domains than treatments targeting amyloid or tau.

## Conclusions

Using the results presented above to select combinations of endpoints for an AD CT, could increase the likelihood of a trial being successful. The methodology presented here has been applied having in mind more traditional clinical trials conducted in the AD area. However, this could also be applied to trials focusing on lifestyle intervention. The results presented here may be particularly applicable to trials such as the FINGER study, where there are no placebo or drug related side effects on the recorded measures. The composite measures examined here could be used to replace the Neuropsychological Test Battery (NTB) measure used in this trial [39].

It would be an interesting question to repeat this analysis with a dataset containing prodromal, and pre-AD subsets, as well as with data where patients were diagnosed using the National Institute on Aging and the Alzheimer’s Association (NIA-AA) criteria [40] to explore whether this diagnostic criteria provides a less variable outcome.

## Electronic supplementary material

Below is the link to the electronic supplementary material.

Supplementary material 1 (DOCX 14 kb)

Supplementary material 2 (DOCX 14 kb)

## List of abbreviations

AD: Alzheimer’s disease
MDES: Minimum detectable effect size
MCI: Mild cognitive impairment
CDRSB: Clinical dementia rating sum of boxes
FDA: U.S Food and Drug Administration
EMA: European Medicines Agency
CT: Clinical trial
MRI: Magnetic resonance imaging
ADAS-Cog: Alzheimer’s Disease Assessment Scale-cognition subscale
ADNI: Alzheimer’s disease neuroimaging initiative
MMSE: Mini-mental state evaluation
MoCA: Montreal cognitive assessment
DMS-VI: Diagnostic and statistical manual
FAQ: Functional Activities Questionnaire
