# Supplementary material for: The importance of endpoint selection: How effective does a drug need to be for success in a clinical trial of a possible Alzheimer’s disease treatment?
Source: Eur J Epidemiol. 2018 Mar 23;33(7):635–44. doi: 10.1007/s10654-018-0381-0 (PMC6061129; doi:10.1007/s10654-018-0381-0)
Supplement: Supplementary file 1 — Supplementary material 1 (DOCX 14 kb) [file 10654_2018_381_MOESM1_ESM.docx]

| **Measure** |  | **0.5** | **1** | **1.5** | **2** | **3** | **4** | **5** | **6** |
| --- | --- | --- | --- | --- | --- | --- | --- | --- | --- |
| ADAS11 | CN | 2.96 | 3.01 | NA | 3.02 | 3.46 | 3.03 | 3.38 | 4.05 |
|  | EMCI | 3.33 | 4.34 | NA | 3.35 | 4.14 | 6.63 | 7.59 | 2.52 |
|  | LMCI | 4.05 | 4.29 | 5.06 | 5.69 | 7.67 | 9.64 | 11.87 | 12.63 |
|  | AD | 4.92 | 6.35 | 20.62 | 8.96 | 11.28 | 5.80 | NA | NA |
| ADAS13 | CN | 3.78 | 3.90 | NA | 3.98 | 4.38 | 4.28 | 4.96 | 5.84 |
|  | EMCI | 4.12 | 5.36 | NA | 4.28 | 5.67 | 8.29 | 9.48 | 2.52 |
|  | LMCI | 5.03 | 5.13 | 6.39 | 7.08 | 9.08 | 11.42 | 13.40 | 14.00 |
|  | AD | 5.16 | 6.87 | 23.13 | 8.99 | 8.22 | 7.32 | NA | NA |
| CDRSB | CN | 0.36 | 0.39 | NA | 0.55 | 0.84 | 0.74 | 1.37 | 1.16 |
|  | EMCI | 0.71 | 0.96 | NA | 1.06 | 1.28 | 1.68 | 2.27 | 2.89 |
|  | LMCI | 1.05 | 1.17 | 1.53 | 2.03 | 2.90 | 3.68 | 4.16 | 4.73 |
|  | AD | 1.56 | 2.30 | 5.78 | 2.88 | 3.39 | 3.34 | NA | NA |
| FAQ | CN | 0.65 | 1.37 | NA | 1.55 | 2.83 | 2.14 | 3.67 | 4.14 |
|  | EMCI | 2.46 | 3.00 | NA | 3.14 | 3.69 | 4.77 | 5.99 | 5.07 |
|  | LMCI | 3.44 | 4.06 | 4.85 | 6.01 | 7.19 | 8.27 | 9.66 | 9.78 |
|  | AD | 4.63 | 5.06 | 7.93 | 5.92 | 5.90 | 8.81 | NA | NA |
| Hippocampus | CN | 273.80 | 258.90 | NA | 286.23 | 279.80 | 354.95 | 389.78 | 482.81 |
|  | EMCI | 204.53 | 231.60 | NA | 302.98 | 338.25 | 408.36 | 197.60 | NA |
|  | LMCI | 216.67 | 229.29 | 264.94 | 352.40 | 389.12 | 521.01 | 463.11 | 567.04 |
|  | AD | 282.29 | 296.15 | NA | 296.85 | NA | NA | NA | NA |
| MMSE | CN | 1.32 | 1.48 | NA | 1.40 | 1.55 | 1.68 | 1.98 | 2.04 |
|  | EMCI | 1.89 | 1.90 | NA | 2.02 | 2.14 | 3.25 | 3.74 | 1.53 |
|  | LMCI | 2.26 | 2.43 | 2.91 | 3.32 | 4.25 | 4.98 | 5.51 | 5.89 |
|  | AD | 3.13 | 3.95 | 6.95 | 5.25 | 7.35 | 4.58 | NA | NA |
| MOCA | CN | 2.35 | 2.39 | NA | 2.50 | 2.65 | 2.76 | 5.66 | NA |
|  | EMCI | 2.70 | 2.61 | NA | 2.85 | 2.89 | 3.56 | 3.33 | 2.65 |
|  | LMCI | 2.38 | 2.25 | NA | 3.24 | 4.40 | 4.77 | 8.76 | NA |
|  | AD | 3.25 | 3.40 | NA | 3.93 | NA | NA | NA | NA |
| Whole Brain | CN | 16777.68 | 18606.62 | NA | 17100.42 | 21856.91 | 27915.03 | 37111.83 | 44980.88 |
|  | EMCI | 16664.65 | 15355.20 | NA | 23312.51 | 18825.93 | 26272.43 | 26509.37 | NA |
|  | LMCI | 17792.18 | 17716.96 | 20651.24 | 23361.71 | 29791.35 | 37729.80 | 43116.69 | 54987.21 |
|  | AD | 15639.47 | 18260.35 | NA | 20928.81 | NA | NA | NA | NA |

**Table S2: Standard deviations of measurements recorded at each timepoint in ADNI, split by diagnostic group at baseline.**
