# Supplementary material for: The importance of endpoint selection: How effective does a drug need to be for success in a clinical trial of a possible Alzheimer’s disease treatment?
Source: Eur J Epidemiol. 2018 Mar 23;33(7):635–44. doi: 10.1007/s10654-018-0381-0 (PMC6061129; doi:10.1007/s10654-018-0381-0)
Supplement: Supplementary file 2 — Supplementary material 2 (DOCX 14 kb) [file 10654_2018_381_MOESM2_ESM.docx]

Table S1: **Participant retention in ADNI by baseline diagnostic group.** Number of participants with data recorded at each time point up to 6 years in the study.

| **CN** | 0 | 0.5 | 1 | 1.5 | 2 | 3 | 4 | 5 | 6 |
| --- | --- | --- | --- | --- | --- | --- | --- | --- | --- |
| ADAS11 | 415 | 398 | 385 | 0 | 359 | 219 | 218 | 111 | 113 |
| ADAS13 | 415 | 395 | 381 | 0 | 358 | 216 | 215 | 111 | 113 |
| MMSE | 415 | 398 | 386 | 0 | 361 | 219 | 218 | 111 | 113 |
| MOCA | 184 | 178 | 174 | 0 | 158 | 31 | 163 | 108 | 113 |
| Hippocampus | 372 | 341 | 330 | 0 | 288 | 132 | 121 | 66 | 70 |
| WholeBrain | 407 | 371 | 356 | 0 | 309 | 156 | 132 | 80 | 87 |
| CDRSB | 415 | 394 | 377 | 0 | 354 | 215 | 212 | 111 | 112 |
| FAQ | 415 | 396 | 384 | 0 | 355 | 216 | 214 | 111 | 112 |
| **EMCI** | 0 | 0.5 | 1 | 1.5 | 2 | 3 | 4 | 5 | 6 |
| ADAS11 | 307 | 275 | 283 | 0 | 246 | 210 | 163 | 75 | 3 |
| ADAS13 | 307 | 275 | 281 | 0 | 242 | 210 | 163 | 73 | 3 |
| MMSE | 308 | 275 | 283 | 0 | 246 | 209 | 165 | 75 | 3 |
| MOCA | 305 | 272 | 281 | 0 | 245 | 206 | 159 | 74 | 3 |
| Hippocampus | 283 | 236 | 247 | 0 | 217 | 77 | 69 | 2 | 0 |
| WholeBrain | 300 | 249 | 258 | 0 | 211 | 73 | 65 | 2 | 0 |
| CDRSB | 308 | 273 | 277 | 0 | 242 | 210 | 159 | 73 | 3 |
| FAQ | 307 | 272 | 278 | 0 | 247 | 206 | 159 | 72 | 3 |
| **LMCI** | 0 | 0.5 | 1 | 1.5 | 2 | 3 | 4 | 5 | 6 |
| ADAS11 | 561 | 535 | 505 | 324 | 431 | 356 | 216 | 128 | 112 |
| ADAS13 | 558 | 532 | 504 | 324 | 428 | 348 | 213 | 127 | 111 |
| MMSE | 561 | 536 | 507 | 325 | 432 | 358 | 215 | 127 | 111 |
| MOCA | 162 | 154 | 149 | 0 | 127 | 109 | 157 | 119 | 105 |
| Hippocampus | 456 | 425 | 388 | 231 | 307 | 154 | 108 | 65 | 54 |
| WholeBrain | 552 | 502 | 476 | 290 | 364 | 204 | 125 | 81 | 73 |
| CDRSB | 561 | 534 | 505 | 321 | 429 | 351 | 216 | 129 | 110 |
| FAQ | 558 | 536 | 506 | 322 | 428 | 359 | 218 | 129 | 112 |
| **AD** | 0 | 0.5 | 1 | 1.5 | 2 | 3 | 4 | 5 | 6 |
| ADAS11 | 338 | 309 | 266 | 4 | 167 | 12 | 3 | 0 | 0 |
| ADAS13 | 332 | 301 | 262 | 4 | 156 | 10 | 3 | 0 | 0 |
| MMSE | 340 | 311 | 267 | 4 | 168 | 12 | 3 | 0 | 0 |
| MOCA | 144 | 121 | 93 | 0 | 27 | 0 | 1 | 0 | 0 |
| Hippocampus | 274 | 219 | 197 | 1 | 109 | 2 | 1 | 0 | 0 |
| WholeBrain | 326 | 264 | 228 | 1 | 131 | 2 | 1 | 0 | 0 |
| CDRSB | 340 | 310 | 267 | 4 | 167 | 12 | 3 | 0 | 0 |
| FAQ | 339 | 310 | 269 | 4 | 170 | 12 | 3 | 0 | 0 |
